# Engineering electrocatalytic activity in nanosized perovskite cobaltite through surface spin-state transition

**DOI:** 10.1038/ncomms11510

**Published:** 2016-05-17

**Authors:** Shiming Zhou, Xianbing Miao, Xu Zhao, Chao Ma, Yuhao Qiu, Zhenpeng Hu, Jiyin Zhao, Lei Shi, Jie Zeng

**Affiliations:** 1 Hefei National Laboratory for Physics Sciences at the Microscale, Hefei Science Center, University of Science and Technology of China, Hefei, Anhui 230026, China; 2 School of Physics, Nankai University, Tianjin 300071, China; 3 State Key Laboratory of Luminescent Materials and Devices, South China University of Technology, Guangzhou 510640, China

## Abstract

The activity of electrocatalysts exhibits a strongly dependence on their electronic structures. Specifically, for perovskite oxides, Shao-Horn and co-workers have reported a correlation between the oxygen evolution reaction activity and the *e*_g_ orbital occupation of transition-metal ions, which provides guidelines for the design of highly active catalysts. Here we demonstrate a facile method to engineer the *e*_g_ filling of perovskite cobaltite LaCoO_3_ for improving the oxygen evolution reaction activity. By reducing the particle size to ∼80 nm, the *e*_g_ filling of cobalt ions is successfully increased from unity to near the optimal configuration of 1.2 expected by Shao-Horn's principle. Consequently, the activity is significantly enhanced, comparable to those of recently reported cobalt oxides with *e*_g_^∼1.2^ configurations. This enhancement is ascribed to the emergence of spin-state transition from low-spin to high-spin states for cobalt ions at the surface of the nanoparticles, leading to more active sites with increased reactivity.

## Abstract (web summary)

The activity of electrocatalysts exhibits a strong dependence on their electronic structures. Here, the authors manipulate the *e*_g_ filling of perovskite cobaltite LaCoO_3_ nanoparticles by changing particle size and show improved oxygen evolution activity with increased numbers of surface high-spin cobalt ions.

## Graphical abstract

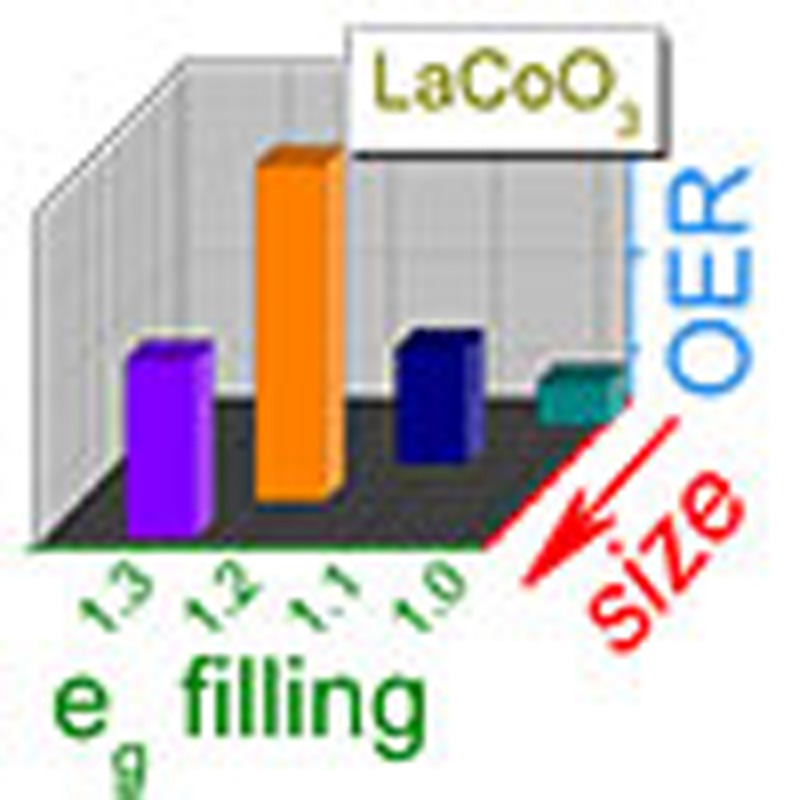

Electrochemical water splitting is regarded as a prime approach for renewable energy conversion and storage^1^,^2^. One of the critical reactions of this process is oxygen evolution reaction (OER). However, this reaction is kinetically sluggish due to a complex multistep four-electron oxidation^1^,^2^,^3^,^4^,^5^. To reach a desirable current density of 10 mA cm^−2^, which is a metric associated with solar fuel synthesis, a considerable overpotential (*η*) relative to thermodynamic potential of the reaction is required, but will hinder the large-scale electrochemical water splitting^3^,^4^. Currently, RuO_2_ and IrO_2_ are among the most active OER catalysts, however, their unacceptable cost and low abundance severely restrict their large-scale applications^5^,^6^. Therefore, it is of great technological and scientific significance to pursue highly efficient alternatives on the basis of earth-abundant nonprecious materials.

Transition-metal oxides and their derivatives have received much attention because of their earth-abundant reserves, low cost, environment-friendly features and remarkable OER activities^7^,^8^,^9^,^10^,^11^,^12^,^13^,^14^,^15^,^16^,^17^,^18^,^19^,^20^,^21^,^22^,^23^. Specially, cobalt oxides, such as layered LiCoO_2_ (refs 11, 12), Co-oxyhydroxides (refs 13, 14), spinel Co_3_O_4_ (refs 15, 16, 17), perovskite Ba_0.5_Sr_0.5_Co_0.8_Fe_0.2_O_3−*δ*_ (BSCF)^18^ and PrBaCo_2_O_5+*δ*_ (ref. 19), have been explored as potential OER catalysts due to their high activities comparable to and even better than the precious metal oxides. Theoretical and experimental works have shown that the OER activity is intrinsically related to the electronic structure of Co ions, including oxidation state and spin configuration, which enlightens the rational design of optimal catalysts^7^,^8^,^9^,^10^,^11^,^12^,^13^,^14^,^15^,^16^,^17^,^18^,^19^,^20^,^21^. For example, Lu *et al*.^12^ recently reported that the OER activity of LiCoO_2_ was remarkably enhanced by tuning the Co oxidation state via electrochemical delithiation process. More interestingly, for perovskite transition-metal oxides, Shao-Horn and co-workers^18^ demonstrated the direct dependence of OER activity on the *e*_g_ orbital filling of the transition-metal ions, and that the oxide BSCF with *e*_g_^∼1.2^ configuration exhibited the highest OER activity. The performance of the BSCF was found to be nearly identical to IrO_2_ in terms of catalytic activity. However, this Co-based oxide undergoes surface amorphization after long-term potential cycles under OER conditions^22^. Therefore, substantial progress is still needed to develop perovskite-type OER catalysts with improved activity and stability. In this respect, Shao-Horn's principle highlights that the optimization of *e*_g_ filling close to 1.2 should be an alternative strategy to develop the transition-metal oxides as the effective OER catalysts. Practically, a recent study by Zhu *et al*.^23^ supported this principle, where an improved OER activity was realized through adjusting the *e*_g_ filling to ∼1.2 with partial substitution of Co by Nb in SrCo_0.8_Fe_0.2_O_3−*δ*_ (ref. 23).

In this work, we present a facile method to modify the electronic structure of Co ions by varying the particle size for the improvement in the OER activity. We focus on the perovskite cobaltite LaCoO_3_ (LCO), which is well-known for its unique thermally-driven transition of Co^3+^ ions from low spin (LS: *t*_2g_^ 6^*e*_g_^ 0^) at low temperatures to higher spin state with *e*_g_ orbital configuration of *e*_g_^∼1.0^ at room temperature^18^,^24^,^25^,^26^,^27^,^28^,^29^,^30^,^31^. This compound was reported to exhibit reasonable OER activity but much less than BSCF^18^. Here, by reducing the particle size, the *e*_g_ filling is successfully increased from unity to close to the optimization configuration of ∼1.2. As a consequence, the OER activity of the 80-nm LCO is higher than those of other sized samples as well as the bulk, and comparable to those of the reported cobalt oxides with *e*_g_^∼1.2^ filling, which enables the nanosized LCO to be applicable as a promising OER catalyst.

## Results

### Crystal structures of the bulk and nanosized LCO

**Figure 1:**
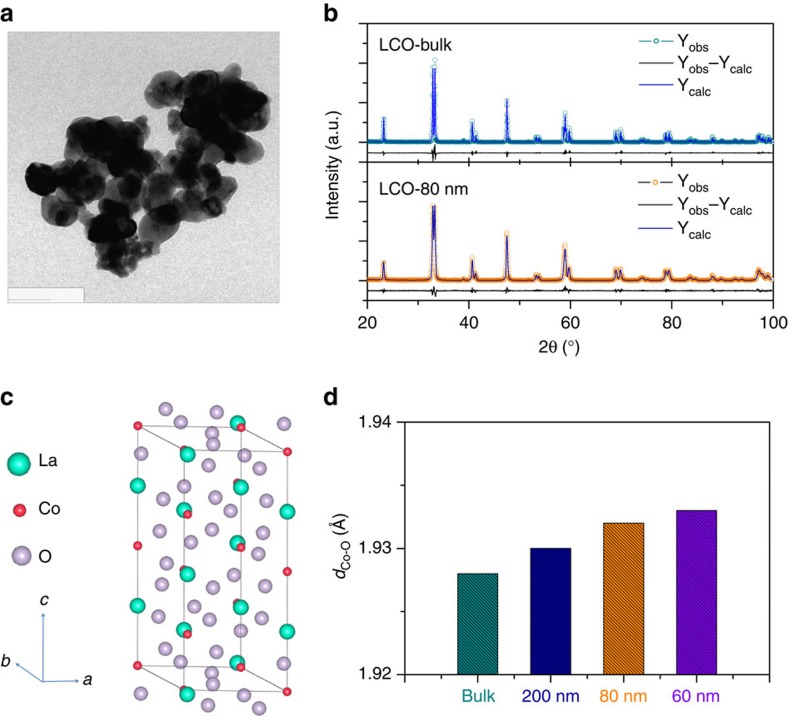
TEM image and crystal structure analyses of the bulk and nanosized LCO. (**a**) TEM image for the 80 nm LCO. (**b**) X-ray diffraction patterns for bulk and 80 nm LCO together with the Rietveld refined results. (**c**) LCO crystal structure. (**d**) The length of Co–O bond for the bulk and nanosized LCO. Scale bar, 180 nm.

The LCO samples were prepared by a sol–gel method^32^,^33^. The precursory powders derived from the gel were annealed at 600, 700 and 800 °C for 6 h to produce the LCO nanoparticles with the particle size of about 60, 80 and 200 nm (ref. 32), respectively, as well as at 1,000 °C for 12 h to the bulk sample with the particle size of about 0.5–1 μm (Supplementary Fig. 1). A representative transmission electron microscopy (TEM) image for the 700 °C annealing sample is shown in Fig. 1a. The high-resolution TEM images and the selective area electron diffraction patterns reveal a single-crystal structure of small LCO particles with a high crystallinity (Supplementary Fig. 2). The X-ray diffraction patterns (Fig. 1b and Supplementary Fig. 3) reveal that all the samples take a rhombohedral structure with R 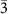
*c* space group (Fig. 1c). The structural parameters obtained from the Rietveld refinements on the diffraction data are given in Supplementary Table 1. As the particle size is reduced, the unit cell is found to be expanded. Specially, the bond length of Co–O exhibits an obvious increase (Fig. 1d).

### Spin structures of the bulk and nanosized LCO

**Figure 2:**
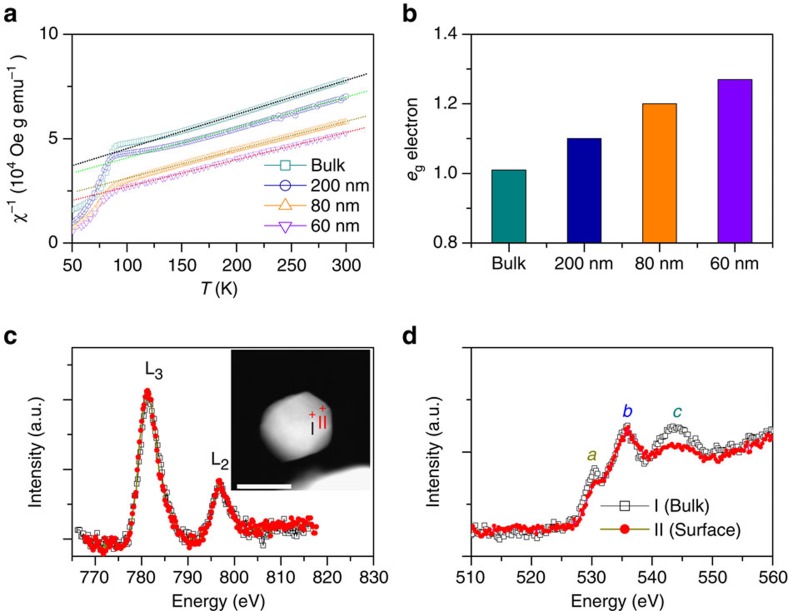
Spin structure analyses of the bulk and nanosized LCO. (**a**) The temperature dependence inverse susceptibilities for all the LCO samples. The dotted lines are the fitting results by a Curie–Weiss law. (**b**) The corresponding *e*_g_ filling. (**c**,**d**) Representative EELS spectra of the 80 nm LCO at Co L-edge and O K-edge, respectively. The inset corresponds to the representative position of EELS acquisition. Scale bar, 50 nm.

The temperature-dependent magnetizations were measured with a magnetic field of *H*=1 kOe under field-cooling procedures for all the samples (Supplementary Fig. 4a) to study the spin structures of Co ions controlled by the particle size. Above 150 K, the susceptibilities derived from the magnetizations (*χ*=*M*/*H*) obey a paramagnetic Curie–Weiss law: 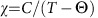, where *C* is Curie constant, and Θ is Curie–Weiss temperature. From the fitting results (Fig. 2a), an effective magnetic moment *μ*_eff_ can be calculated through 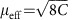 *μ*_B_ (Supplementary Fig. 4b). For the bulk LCO, although the exact nature of whether the higher spin state of Co^3+^ ions at room temperature is intermediate-spin (IS: *t*_2g_^ 5^*e*_g_^ 1^) state or a mixture of LS and high spin (HS: *t*_2g_^ 4^*e*_g_^ 2^) states was controversial in the past decades^24^,^25^,^26^,^27^, a large number of recent theoretical and experimental studies reveal that the mixture of LS and HS states is more favourable^28^,^29^,^30^,^31^. Here, the calculated *μ*_eff_ of 3.48 *μ*_B_ for the bulk sample is well consistent with those values reported for the polycrystalline bulk LCO, which corresponds to the Co^3+^ ions in 50% HS+50% LS states as well as the *e*_g_ filling of ∼1.0 (refs 25, 28, 29, 30, 31). For the nanosized LCO, *μ*_eff_ shows a gradual increase with the decrease of the particle size, suggesting that the spin state of partial Co ions transmits from LS to HS state. Using the calculated *μ*_eff_, the spin states are estimated to be 55% HS+45% LS, 60.5% HS+39.5% LS and 63.7% HS+36.3% LS for the 200, 80 and 60 nm samples (Supplementary Note 1 and Supplementary Table 2), meaning that about 5, 10.5 and 13.7% Co^3+^ ions in LS state change to be in HS state, respectively. As shown in Fig. 2b, the corresponding *e*_g_ filling is about 1.1, 1.2 and 1.27 for the nanosized LCO, respectively. It is worthwhile to emphasize that by reducing the particle size to about 80 nm, we successfully tune the *e*_g_ filling from unity to the optimization value of 1.2 expected by Shao-Horn's principle.

To further confirm the spin-state transition and to explore its possible origin, the electron energy loss spectroscopy (EELS) analyses on the LCO nanoparticles were performed on the scanning TEM. Figure 2c,d shows the representative EELS spectra at Co L- and O K-edges from the centre and edge of the 80 nm nanoparticle, which are sensitive to the electronic structure from the core (bulk) and shell (surface), respectively^34^,^35^. For the Co L-edge spectra, no noticeable changes in the L_3_/L_2_ ratio between the bulk and surface were found, suggesting that the oxidation state of Co ions remained unchanged^35^,^36^,^37^,^38^,^39^. For the O K-edge spectra, three characteristic peaks near the edge onset, labelled *a*, *b* and *c*, were observed. The prepeak *a*, *b* and *c* were assigned to the hybridization of O 2*p* with Co 3*d*, La 5*d* and Co 4*sp* orbitals, respectively^36^,^37^,^38^,^39^. Compared with that from the centre position, the spectrum from the edge shows an obvious reduction in the intensity of the prepeak *a*. Similar results are also found in the other nanoparticles (Supplementary Fig. 5). This reduction is generally attributed to the formation of oxygen vacancy or the weakening of Co 3*d*–O 2*p* hybridization^36^,^37^,^38^,^39^. Since the Co L-edge spectra revealed no change in the Co oxidation state, the formation of oxygen vacancies can be excluded. Thus, the modification of O K-edge would originate from the change of the Co 3*d*–O 2*p* hybridization. Previous works have been widely reported that the spin-state transition of Co^3+^ ions in the LCO can significantly modify the hybridization of Co 3*d*–O 2*p* orbitals and then the intensity of the prepeak *a* (refs 35, 36, 37, 38, 39). In the LS configuration, the 3*d e*_g_ levels of Co^3+^ ions are completely empty, allowing the electrons from the filled O 2*p* levels to be shared with Co *e*_g_ orbitals, and accordingly creating O 2*p* holes. Thus, the hybridization of Co 3*d* with the O 2*p* states promotes electron transitions between 1*s* and the unfilled O 2*p* state, resulting in the prepeak *a* of the O K-edge. However, as a HS state of the Co^3+^ ions emerges, the *e*_g_ orbitals are increasingly occupied, which prevents the charge transfer and weakens the hybridization of O 2*p* with Co 3*d* orbitals, resulting in the decreased intensity of the prepeak *a* (refs 36, 37, 38, 39). Therefore, the decrease in the intensity of the prepeak *a* at the surface confirms the existence of surface spin-state transitions in the nanosized LCO.

We proposed a mechanism to explain the presence of surface spin-state transitions of Co^3+^ ions where the modified crystal field splitting of Co 3*d* orbital at the surface favors the Co^3+^ ions to be in HS states, which has also been reported in nanosized stoichiometric LiCoO_2_ (ref. 40). Assuming that Co^3+^ ions within surface layers are all transited to be in HS state, we can give a rough estimate of the *e*_g_ filling for the nanosized LCO on the basis of a simple core-shell model (Supplementary Note 2 and Supplementary Fig. 6). For nanosized perovskite oxides with the particle size ranging from tens to hundreds of nanometres, the surface layers are usually reported to be about 2–5 nm in the thickness^41^,^42^,^43^,^44^. Taking the thickness of 3 nm (see Supplementary Fig. 2), the estimated volume fractions of the surface layer are about 8.7, 20.8 and 27.1% for the 200, 80 and 60 nm LCO, respectively. Consequently, the increased fractions of the HS Co^3+^ ions are 4.4, 10.4 and 13.6%, which means that the *e*_g_ fillings are increased to be about 1.09, 1.21 and 1.27, respectively. These values are well consistent with those obtained from the magnetizations, further supporting that the tuning of *e*_g_ filling by the size reduction originates from the surface spin-state transition. In addition, since the radii of Co^3+^ ions increases when their spin state changes from LS to HS, the presence of this transition is also confirmed by the crystal structure data, where the expansion of the unit cell and the increase of Co–O length under decreasing the particle size are found (Supplementary Table 1 and Fig. 1d).

### OER activities of the bulk and nanosized LCO

**Figure 3:**
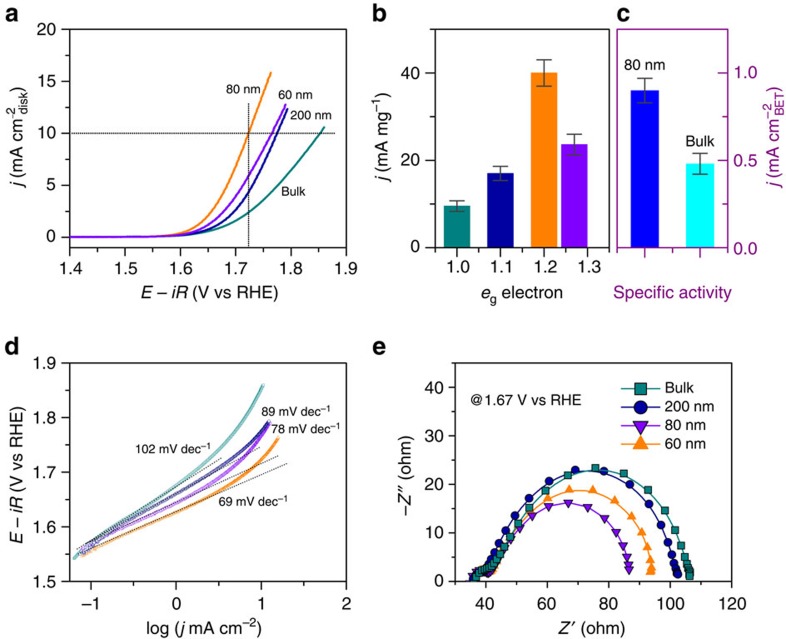
OER activities of the bulk and nanosized LCO. (**a**) Polarization curves of the bulk and nanosized LCO. (**b**) Mass and (**c**) special activities at *η*=0.49 V. (**d**) Tafel plots for the bulk and nanosized LCO. (**e**) Nyquist plots for the bulk and nanosized LCO. Error bars represent the s.d. from at least three independent measurements.

To shed light on the role that the surface spin-state transition plays in the OER activity for the LCO, the electrochemical measurements were carried out in O_2_-saturated 0.1 M KOH solutions using a standard three-electrode system. Figure 3a shows the *iR*-corrected polarization curves for all the samples, where the LCO nanoparticles exhibited smaller onset overpotentials than that of the bulk (∼0.37 V). In particular, the smallest onset overpotential of ∼0.32 V was observed in the 80 nm LCO. Similarly, the overpotential *η* required to achieve a current density of 10 mA cm^−2^ was also reduced from 0.62 V for the bulk to 0.54, 0.49 and 0.55 V as the particle size decreased to about 200, 80 and 60 nm, respectively. Figure 3b plots the dependence of mass activity at *η*=0.49 V on the *e*_g_ filling. As the *e*_g_ filling approached the optimal configuration of ∼1.2, the current density reached the largest value, which was about 4.3 times larger than that of the bulk. Moreover, the corresponding Tafel plots (Fig. 3d) also reveal that the 80 nm LCO possessed the smallest Tafel slope of ∼69 mV dec^−1^, much smaller than that of the bulk (∼102 mV dec^−1^). This large reduction suggests that the rate-determining step tends to change from the –OH adsorption to the O–OH formation^18^,^45^,^46^. In addition, the preliminary stability tests for the bulk and 80 nm LCO under a constant galvanostatic current of 10 A g^−1^ (Supplementary Fig. 7) also demonstrate that the 80 nm sample exhibited better stability than the bulk. As revealed by Co 2*p* X-ray photoelectron spectra of the 80 nm LCO before and after the electrolysis (Supplementary Fig. 8), no visible changes for the Co 2*p* spectra were found during the electrolysis, suggesting that the electronic state of the Co ions may remain unchanged. All of the above results clearly indicate that the OER activity of the LCO is successfully modified by controlling the particle size. As the particle size is reduced to about 80 nm with the *e*_g_^∼1.2^ configuration, the activity is significantly enhanced.

### The origin of the enhanced OER activity

As the sample size is reduced, the enhanced mass catalytic activity towards the OER is largely ascribed to the increase of the surface area. Such scenarios were reported in various Co-based oxides such as BSCF^18^, SrNd_0.1_Co_0.7_Fe_0.2_O_3−*δ*_ (ref. 23) and NiCo_2_O_4_ (ref. 47). However, in those cases, the size reduction leaded to a large decrease in the specific OER activities, that is, the normalized activities by the surface area. Since the specific activity reflects the intrinsic activity of the catalysis, this decrease indicates that intrinsic OER activity was deteriorated by reducing the sample size for those oxides. To clarify whether the enhancement of the OER activity in our LCO nanoparticles is intrinsic, the specific activities are further calculated on the basis of two types of surface areas, the Brunauer-Emmett-Teller (BET) surface areas and the electrochemically active surface areas, obtained by means of the gas desorption (Supplementary Fig. 9) and electrochemical double-layer capacitance measurements (Supplementary Fig. 10 and Supplementary Note 3), respectively. The specific activities at *η*=0.49 V normalized by the surface areas exhibit similar dependences on the *e*_g_ filling to the mass activity (Supplementary Fig. 11). Compared with the bulk, the 80 nm sample is still 1.8 times more active in the specific activity normalized by the BET area (Fig. 3c), which strongly suggests that the increased number of active sites from the surface areas may be not the main contribution to the significant enhancement of the OER activity. The improved performance would be mainly attributed to the increased reactivity of the active sites due to the spin-state transition of Co^3+^ ions at surfaces. When the *e*_g_ filling of Co^3+^ ions increases from about 1.0 to 1.2, the electron occupancy of the Co 3*d*–O 2*p* σ* band increases with the elongation of the length of Co–O bond as shown in Figs 1d and 2d. Thus, the hybridization of Co 3*d*–O 2*p* orbitals and the strength of Co–O bond become weaker, which leads to a less surface coverage by –OH groups on the active sites and thereby facilitates the formation of –OOH species^45^,^46^. As a result, the Tafel slope is reduced and the OER activity is improved. On the other hand, it has been generally demonstrated that H_2_O molecules are initially adsorbed onto the surface of catalysts during the OER process^13^,^18^,^45^. Consequently, the adsorption energy of H_2_O onto the active site plays a crucial role in the OER activity. Our density functional theory (DFT) calculations on the adsorption energy of H_2_O onto the surface Co ions in different spin states reveal that the surface Co ions being in HS state are more favourable for adsorbing H_2_O molecular (Supplementary Fig. 12 and Supplementary Table 3), well consistent with the improvement of the OER activity by reducing the particle size to 80 nm. However, it is worthwhile to note that as the particle size is further reduced to about 60 nm the activity decreases again, which cannot be explained by the above factors. We propose that the excessive *e*_g_ occupancy (>1.2) of the Co^3+^ ions in this sample would make the charge transfer ability lower. As such, when the two neighboured Co ions in Co–O–Co network are both in HS state, the half-filling of *e*_g_ orbitals tends to prevent the charge transfer. To confirm this point, the electrochemical impedance spectroscopy experiments have been carried out. As shown in Fig. 3e, the Nyquist plots reveal that the charge transfer resistance gradually decreases with the reduction of the particle size to 80 nm, while increases again as the size is further reduced to 60 nm. Therefore, we conclude that the modifications of the Co–O binding strength and the charge transfer ability associated with the surface spin-state transition in the LCO are responsible for the size-dependent OER activity.

Finally, we compared the OER activity of our LCO samples with those of the recently reported Co-based perovskite oxides with the optimal configuration of *e*_g_^∼1.2^. As illustrated in Table 1, it is interesting to find that the 80 nm LCO exhibits a well comparable activity with those well-known catalysts, which further consolidates Shao-Horn's principle and suggests that tuning the spin state can provide an effective strategy to improve the OER activity.

## Discussion

In summary, we highlight an effective strategy to engineer the electronic configuration of perovskite cobalt oxide for the development of high active electrocatalysts. By reducing the particle size to about 80 nm, the spin filling of Co ions in LCO is successfully tuned from unity (bulk) to near the optimization configuration of ∼1.2 expected by Shao-Horn's principle. Through X-ray diffraction, magnetic measurements and EELS analysis, we confirm that this modification originates from the size-induced spin-state transition of Co^3+^ ions from LS to HS state. Consequently, the nanosized sample exhibits an improved OER activity with lower overpotential, smaller Tafel slope and better stability compared with the bulk. More interestingly, the performance of the 80 nm LCO can be comparable with those of the reported cobalt oxides with *e*_g_^∼1.2^ filling, suggesting that the LCO in this nanosized form can serve as a promising OER catalyst. Our work paves the way for the rational design of high-efficient OER catalysts.

## Methods

### Synthesis and characterization

La(NO_3_)_3_·6H_2_O and Co(NO_3_)_2_·6H_2_O were dissolved in deionized water, followed by the addition of a mixture of citric acid and ethylene glycol. Subsequently, the obtained transparent solution was slowly evaporated to get a gel, which was decomposed at about 400 °C for 4 h to result in dark brown powders. The precursor powders were further annealed at 600, 700 and 800 °C for 6 h to produce LCO nanoparticles with different particle sizes, and at 1,000 °C for 12 h to the bulk sample. The phase purity and crystal structure of the samples were determined by X-ray diffraction at room temperature on a Rigaku TTR-III diffractometer using Cu K_a_ radiation (*λ*=1.5418 Å). The field emission SEM and TEM images were obtained on a JEOL-2010 SEM and a JEM-2100F TEM, respectively. The HRTEM images and the EELS analyses were performed on a JEOL JEM-ARM200F TEM/scanning TEM with a spherical aberration corrector. The magnetic measurements were carried out with a MPMS SQUID magnetometer. The nitrogen adsorption−desorption isotherms were conducted on a Micromeritics ASAP 2000 system at 77 K. X-ray photoelectron spectra were carried out on an ESCALAB 250 X-ray photonelectron spectrometer with Al Kα as the excitation source.

### Electrochemical measurements

The electrochemical tests were performed in O_2_-saturated 0.1 M KOH with a conventional three-electrode on the CHI660B electrochemical station. Saturated Ag/AgCl and platinum wires were used as the reference and the counter electrodes, respectively. The reference electrode was calibrated with respect to the reversible hydrogen electrode (RHE), which was carried out in the high-purity hydrogen saturated electrolyte with a Pt wire as the working electrode. Cyclic voltammetry was run at a sweep rate of 1 mV s^−1^. The average of the two potentials at which the current crossed zero was taken to be the thermodynamic potential for the hydrogen electrode reactions. In 0.1 M KOH, *E*_RHE_=*E*_Ag/AgCl_+0.964 V. To prepare the working electrode, 3.5 mg of electrocatalyst and 20 μl of 5 wt% Nafion solutions were dispersed in 1 ml ethanol with sonication for at least 30 min to form a mixted ink. Then, 5 μl of this solution was drop-casted onto a 3 mm in diameter glassy carbon electrode and dried naturally, yielding a catalyst loading of 0.25 mg cm^−2^. Linear sweeping voltammograms were obtained at a scan rate of 5 mV s^−1^. The potentials are corrected to compensate for the effect of solution resistance, which were calculated by the following equation: *E*_*iR*−corrected_=*E*−*iR*, where *i* is the current, and *R* is the uncompensated ohmic electrolyte resistance (∼36 Ω) measured via high frequency ac impedance in O_2_-saturated 0.1 M KOH. The polarization curves were replotted as overpotential (*η*) vs log current (log *j*) to get Tafel plots for quantification of the OER activities of investigated catalysts. Electrochemical impedance spectroscopy were conducted with AC voltage with 5 mV amplitude at the potential of 1.67 V vs RHE within the frequency range from 100 KHz to 100 mHz. Durablity test was performed at room temperature under a constant galvanostatic current of 10 A g^−1^. Error bars represented s.d. from at least three independent measurements.

### DFT calculations

DFT+U calculations with the Vienna *ab initio* simulation package^48^ for a water molecule adsorbed on (001) surface of LCO with LS and HS state on Co^3+^ ions were performed to study the influence on adsorption energy of water molecule with different spin states of Co^3+^ ions. A slab model consisting of eight atom-layers (La_16_Co_16_O_48_) was used to simulate the (001) surface with two terminations. In calculations, an effective U of 3.4 eV was added on Co 3*d* orbital, the plane wave energy cut-off was set to 400 eV, and a 2 × 2 × 1 Monkhorst–Pack *k*-point mesh was used. During geometry optimization, converge criteria were 10^−5^ eV for energy and 0.05 eV Å^−1^ for force. The LS of Co^3+^ was not obtained on CoO_2_ terminated (001) surface, since surface Co^3+^ was in a five-coordinated structure and turns into HS during the calculation even though it was set to LS initially.

### Data availability

The data that support the findings of this study are available from the corresponding author on request.

## Additional information

**How to cite this article:** Zhou, S. *et al*. Engineering electrocatalytic activity in nanosized perovskite cobaltite through surface spin-state transition. *Nat. Commun.* 7:11510 doi: 10.1038/ncomms11510 (2016).

## Supplementary Material

Supplementary Information Supplementary Figures 1-12, Supplementary Tables 1-3, Supplementary Notes 1-3 and Supplementary References

## Figures and Tables

**Table 1:** Comparison of OER activity for different catalysts.

**Catalyst**	**Onset overpotential (V vs RHE)**	***η*** **@** ***j*****=10 mA cm**^−2^ **(V)**	**Tafel slope (mV dec^−1^)**	***e***_**g**_ **filling**	**Ref.**
LCO-bulk	0.37	0.62	102	*e*_g_^∼1.0^	This work
LCO-80 nm	0.33	0.49	69	*e*_g_^∼1.2^	This work
Ba_0.5_Sr_0.5_Co_0.8_Fe_0.2_O_3−*δ*_	—	0.49	84	*e*_g_^∼1.2^	(ref. 21)
Ba_0.5_Sr_0.5_Co_0.8_Fe_0.2_O_3−*δ*_	0.30	0.50	94	*e*_g_^∼1.2^	(ref. 23)
SrCo_0.9_Ti_0.1_O_3−*δ*_	—	0.51	88	*e*_g_^∼1.16^	(ref. 21)
SrNb_0.1_Co_0.7_Fe_0.2_O_3−*δ*_	0.30	0.50	76	*e*_*g*_^∼1.2^	(ref. 23)

OER, oxygen evolution reaction.
