# Supplementary material for: Engineering electrocatalytic activity in nanosized perovskite cobaltite through surface spin-state transition
Source: Nat Commun. 2016 May 17;7:11510. doi: 10.1038/ncomms11510 (PMC4873645; doi:10.1038/ncomms11510)
Supplement: Supplementary Information — Supplementary Figures 1-12, Supplementary Tables 1-3, Supplementary Notes 1-3 and Supplementary References [file ncomms11510-s1.pdf]

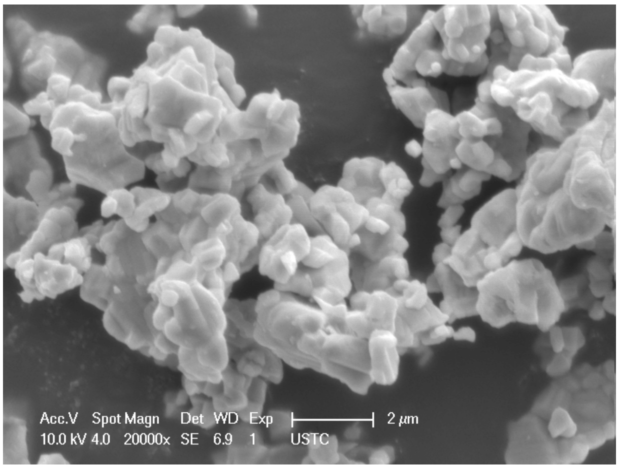

Supplementary Figure 1 | SEM image for the bulk LCO.

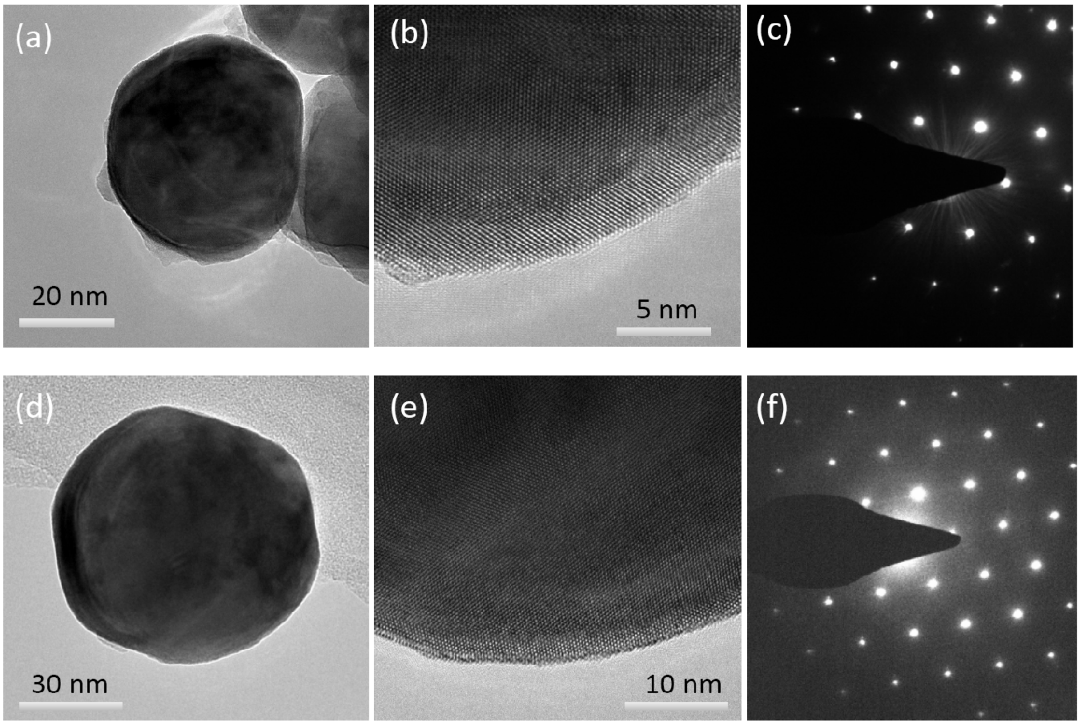

Supplementary Figure 2 | TEM and HRTEM images of LCO nanoparticles. (**a**)-(**c**) TEM, HRTEM images, and SAED pattern for the 60 nm LCO, respectively. (**d**)-(**f**) TEM, HRTEM images, and SAED pattern for the 80 nm LCO, respectively.

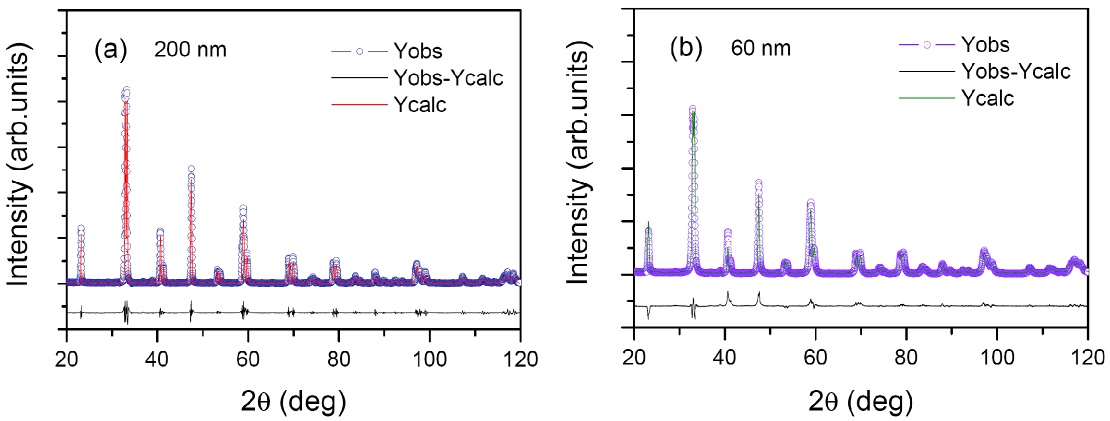

Supplementary Figure 3 | Structure of LCO nanoparticles. (**a**) and (**b**) The XRD patterns together with the Rietveld refinements for the 200 and 60 nm LCO, respectively.

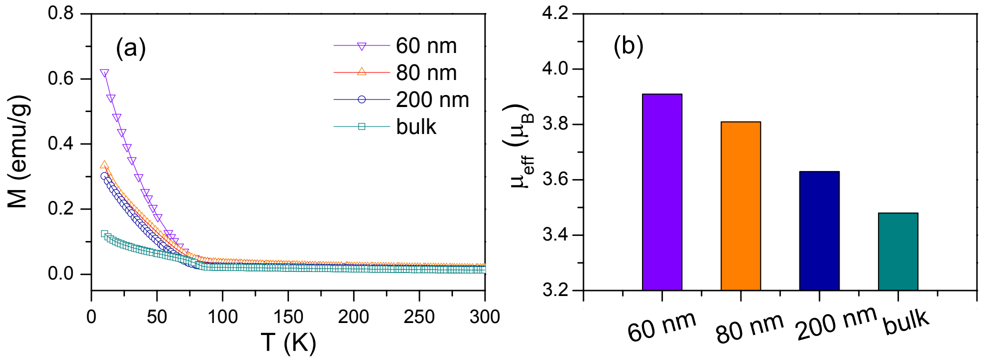

Supplementary Figure 4 | Magnetic properties of the bulk and nanosized LCO. (**a**) Temperature dependent magnetization under H = 1 kOe and (**b**) the calculated effective magnetic moment for the nanosized and bulk LCO.

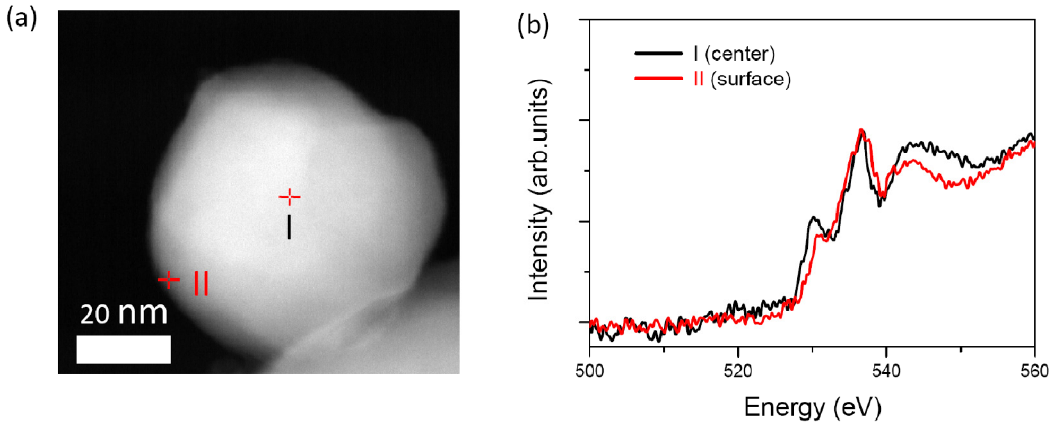

Supplementary Figure 5 | EELS of LCO nanoparticles. (**a**) Representative positions of EELS acquisition and (**b**) the corresponding EELS spectra for 60 nm LCO at O-K edge.

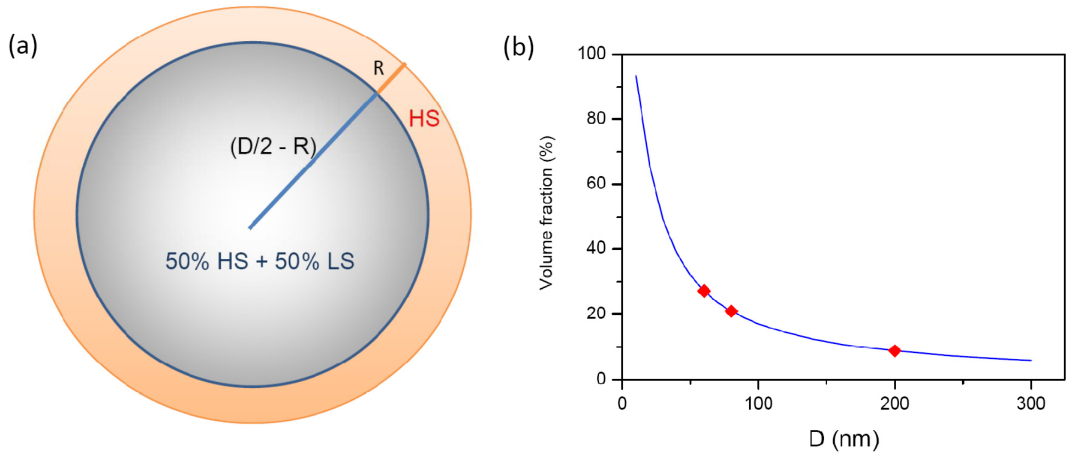

Supplementary Figure 6 | Core-shell model. (**a**) Core-shell model for the spin configurations of LCO nanoparticles. (**b**) Particle size-dependent volume fraction of shell (surface) layer for LCO nanoparticles.

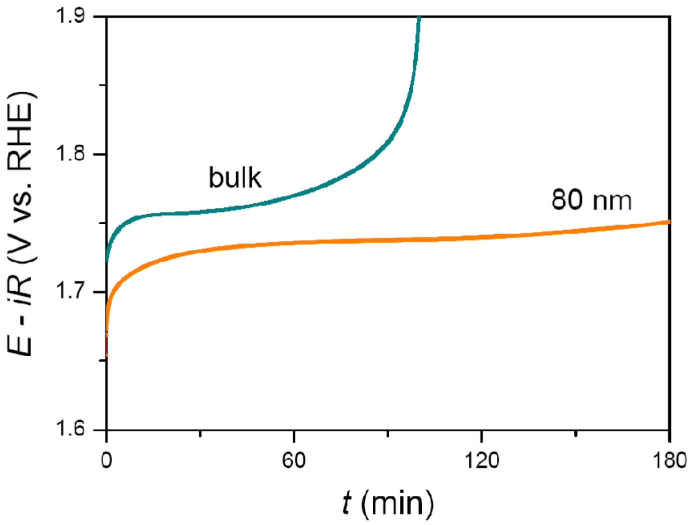

Supplementary Figure 7 | Long-term stability tests. Chronopotentiometric measurements at J = 10 A g^{-1} for the bulk and 80 nm LCO.

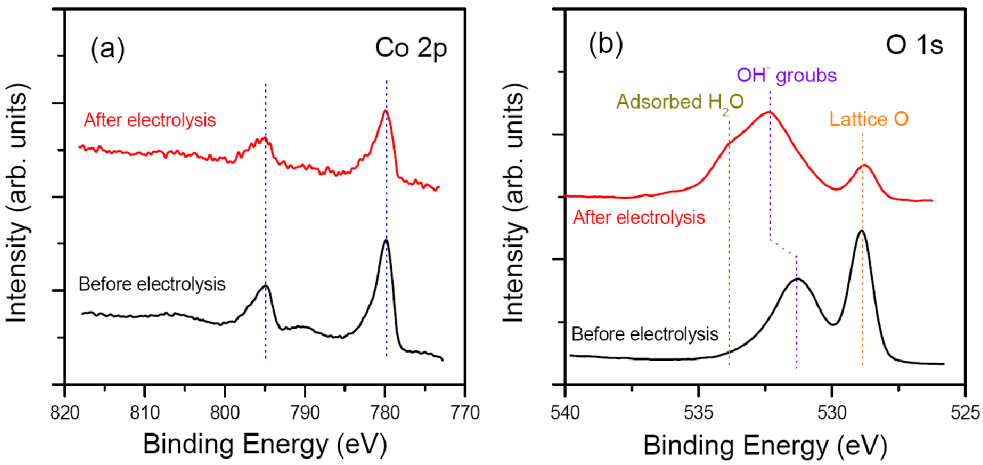

Supplementary Figure 8 | XPS spectra. (**a**) Co 2p and (**b**) O 1s spectra before and after the durability tests for the 80 nm LCO. During the tests, no visible change can be found in the Co 2p spectra, whereas the oxygenated surface species can be found in O 1s spectra.

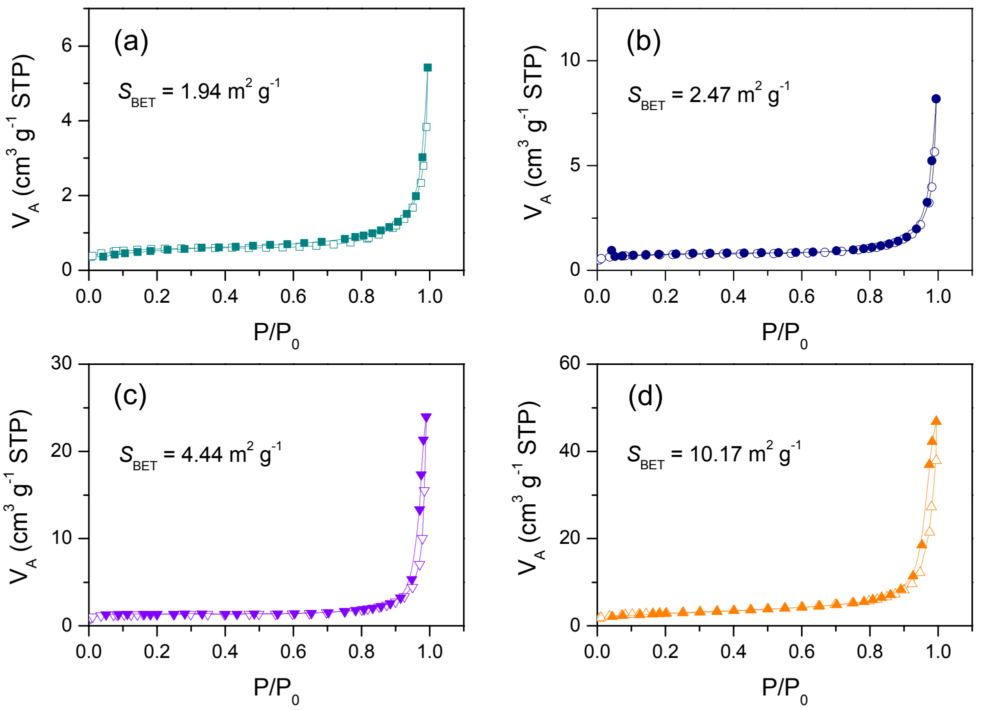

Supplementary Figure 9 | (a)-(d) Nitrogen adsorption-desorption isotherm curves for the bulk, 200, 80, and 60 nm LCO, respectively.

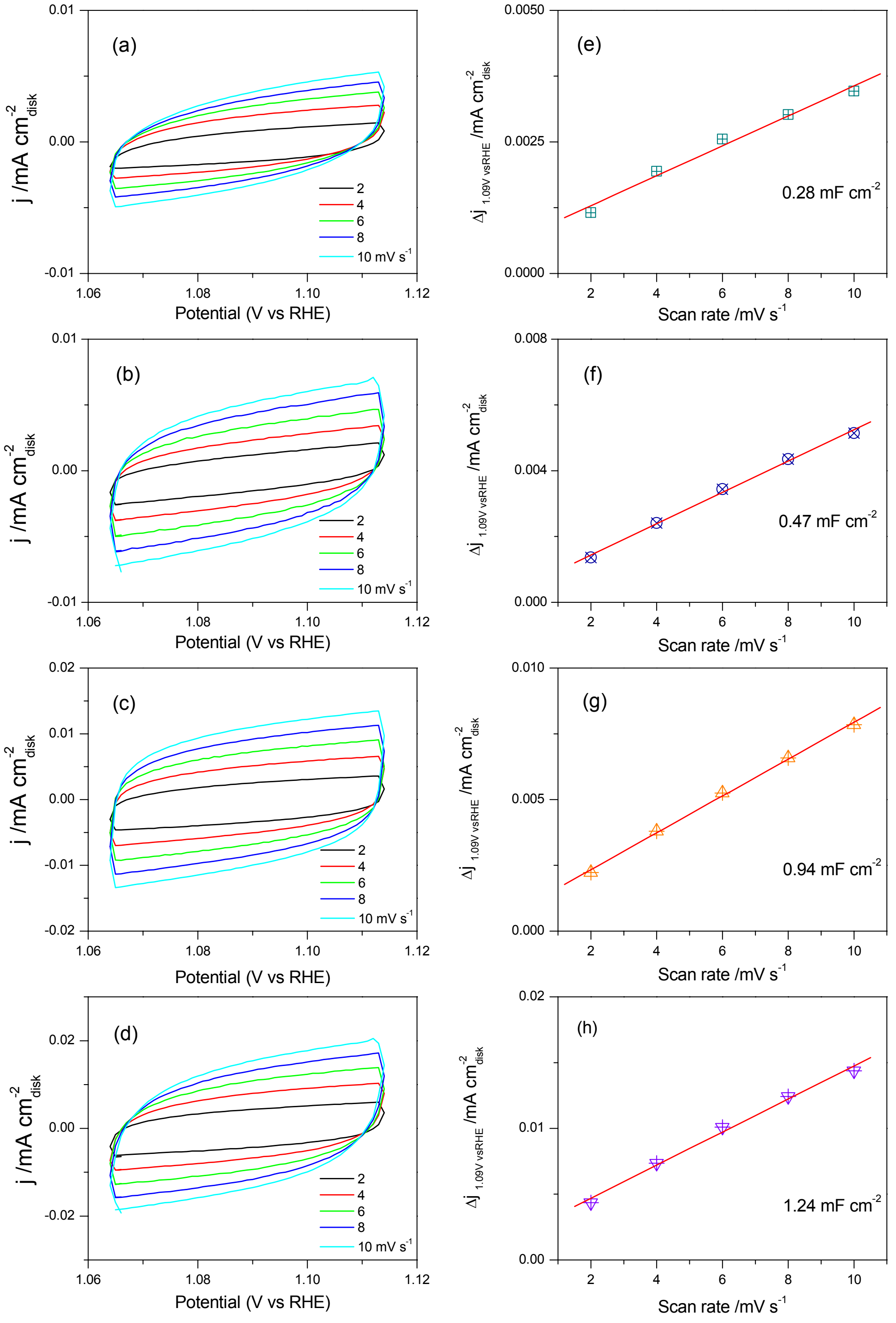

Supplementary Figure 10 | ECSA analyses of the bulk and nanosized LCO. (**a**)-(**d**) CVs at different scan rates of in a potential window where no Faradaic processes occur (1.06-1.12 V vs. RHE) for the bulk, 200, 80 and 60 nm LCO, respectively. (**e**)-(**h**) Charging current density differences ($\Delta J = (J_+ - J_-)/2$) plotted against scan rates for the bulk, 200, 80 and 60 nm LCO, respectively. The linear slope is equivalent to the double-layer capacitance (C_{dl}).

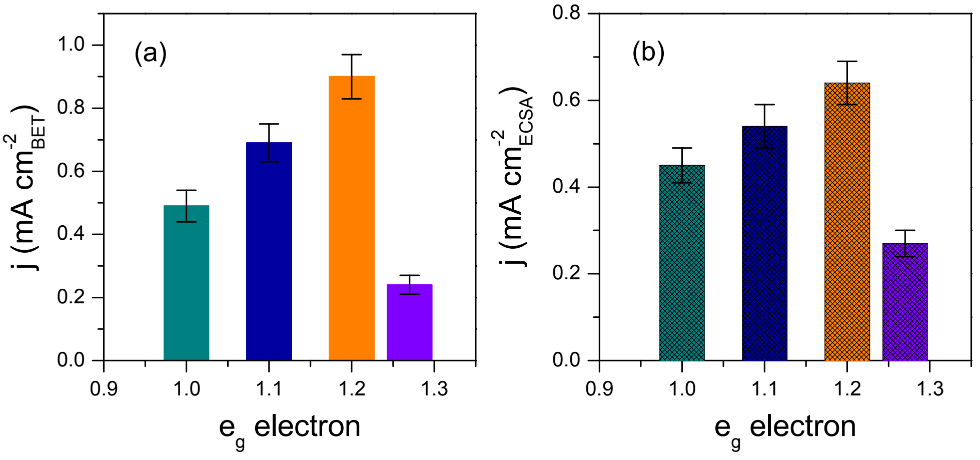

Supplementary Figure 11 | OER specific activity as a function of e_g electron filling. (**a**) and (**b**) OER specific activities at η = 0.49 V by normalized with the BET area and ECSA for the LCO with different e_g filings, respectively. Error bars represent s.d. from at least three independent measurements.

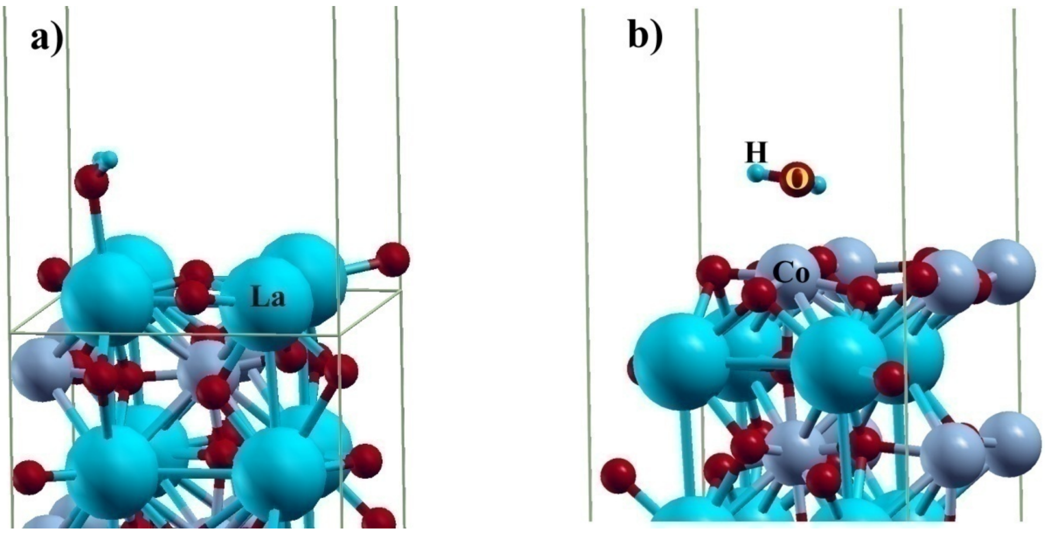

Supplementary Figure 12 | Model of water adsorbed on LCO surface. (**a**) Water adsorbed on top of La on LaO-terminated (001) surface, and (**b**) water adsorbed on top of Co on CoO_2-terminated (001) surface. Light green frames represent the periodic boundaries.

Supplementary Table 1 | Lattice parameters *a* and *c*, volume of the unit cell (*V*), x_O for oxygen site position (*x*,0,1/4) , and agreement factors *R* for LCO samples from the Rietveld refinement.

	**LCO-bulk**	**200 nm**	**80 nm**	**60 nm**
**Space group**	$R\bar{3}c$	$R\bar{3}c$	$R\bar{3}c$	$R\bar{3}c$
**a** (Å)	5.4416(1)	5.4423(1)	5.4422(2)	5.4436(2)
**c** (Å)	13.0889(1)	13.0999(1)	13.1093(4)	13.1246(6)
**V** ($Å^3$)	335.649(5)	336.105(14)	336.254(18)	336.819(21)
$\mathbf{x_O}$	0.5445(9)	0.5469(12)	0.5501(11)	0.5490(12)
$\mathbf{R_{Bragg}}$	3.48%	4.50%	4.84%	10.6%
$\mathbf{R_{wp}}$	12.8%	13.6%	14.6%	14.8%

Supplementary Table 2 | Effective magnetic moment (μ_{eff}) and e_g orbital occupation for different spin states of Co^{3+} ions in $LaCoO_3$ (LS: low spin; HS: high spin).

Spin state	LS	HS	50% HS + 50% LS	55% HS + 45% LS	60.5% HS +39.5% LS	63.7% HS + 36.3% LS
μ_{eff} (μ_B)	0	4.90	3.46	3.63	3.81	3.91
e_g occupation	e_g^{0}	e_g^{2}	$e_g^{\sim1.0}$	$e_g^{\sim1.1}$	$e_g^{\sim1.2}$	$e_g^{\sim1.27}$

Supplementary Table 3 | Adsorption energy and oxygen-metal distance for water molecule adsorbed on $LaCoO_3$ (001) surfaces with LS and HS surface Co^{3+} (minus for exothermic).

Termination	Adsorption energy (eV)		Oxygen-metal distance (Å)	
	LS	HS	LS	HS
LaO (001)	0.28	-0.10	2.83	2.78
CoO_2 (001)	-	-0.56	-	2.22

Supplementary Note 1. The temperature dependent magnetizations (M) for the nanosized and bulk LCO under $H = 1$ kOe are shown in Supplementary Fig. 2a. The effective magnetic moments (μ_{eff}) for all the samples are obtained by $\mu_{eff} = \sqrt{8C}\mu_B$, where C is Curie constant and obtained from the fittings on the susceptibility ($\chi = M/H$) above 150 K by a Curie-Wiess law (see Main Text). The obtained values are plotted in Supplementary Fig. 2b. Using these values, the volume fractions of Co ions in HS and LS states can be calculated from the relationship: $\mu_{eff} = g\mu_B\sqrt{S_{HS}(S_{HS}+1)V_{HS}+S_{LS}(S_{LS}+1)V_{LS}}$, where S_{HS} (= 2) and S_{LS} (=0) are the S values, and V_{HS} and V_{LS} (= 1 - V_{HS}) are the volume fractions for Co^{3+} ions in HS and LS states, respectively. Consequently, the e_g electron (x) can be further calculated by $x = S_{HS}$ x V_{HS} + S_{LS} x V_{LS} = 2 V_{HS}. The calculated results are shown in Supplementary Tab. 2.

Supplementary Note 2. A simple core-shell model is shown in Supplementary Fig. 3a. The volume fraction of the shell (surface) layer is roughly estimated by: V_S (%) = 100 x [1- (1-2R/D)^3], where R and D is the average thickness of surface layer and the diameter of the particle (simplified as a ball), respectively. Using the thickness of surface layer R = 3 nm, the dependence of volume fraction of surface layer (V_S) with particle size (D) is shown in Supplementary Fig. 3b. Assuming that the spin states of Co^{3+} ions in the core remain in 50% HS + 50 % LS and that in the shell is in 100% HS, the e_g electron (x) can be roughly estimated by: x = (1 –$V_S/100$) x (50% x S_{HS} + 50% x S_{LS}) + $V_S/100$ x 100% x S_{HS} = 1 + $V_S/100$. Based on this model, the estimated e_g fillings are about 1.09, 1.21, and 1.27 for the 200, 80, and 60 nm LCO, which are close to those values obtained from the magnetization (Supplementary Tab. 2).

Supplementary Note 3. The ECSA was determined by measuring the capacitive current associated with double-layer charging from the scan-rate dependence of cyclic voltammetry (CV)[1-3]. For this, the potential window of CV was 1.06 -1.12 V vs. RHE. The scan rates were 2, 4, 6, 8, and 10 mV s^{-1}. The double layer capacitance (C_{dl}) was estimated by plotting the Δ J = (J_+ - J_-)/2 at 1.09 V vs. RHE against the scan rate. The linear slope is C_{dl}, which is about 0.28, 0.47, 0.94 and 1.24 mF cm^{-2} for the bulk, 200, 80, and 60 nm LCO, respectively. Using a value of 60 μF cm^{-2} as a common estimate for oxide surfaces[1,4] and a catalyst loading of 0.25 mg cm^{-2}, the ECSA is estimated to be about 1.9, 3.1, 6.3 and 8.3 m^2 g^{-1} for the bulk, 200, 80, and 60 nm LCO, respectively.

Supplementary References

1. Hong, W. T., Risch, M., Stoerzinger, K. A., Grimaud, A., Suntivich, J. & Shao-Horn, Y. Toward the rational design of non-precious transition metal oxides for oxygen electrocatalysis. *Energy Environ. Sci.* **8**, 1404-1427 (2015).
2. Merki, D., Vrubel, H., Rovelli, L., Fierro, S. & Hu, X. L. Fe, Co, and Ni ions promote the catalytic activity of amorphous molybdenum sulfide films for hydrogen evolution. *Chem. Sci.* **3**, 2515-2525 (2012).
3. Bao, J., Zhang, X. D., Fan, B., Zhang, J. J., Zhou, M., Yang, W. L., Hu, X., Wang, H., Pan, B. C. & Xie, Y. Ultrathin spinel-structured nanosheets rich in oxygen deficiencies for enhanced electrocatalytic water oxidation. *Angew. Chem. Int. Ed.* **127**, 7507–7512 (2015).
4. Bockris, J. O. & Otagawa, T. The electrocatalysis of oxygen evolution on perovskites. *J. Electrochem. Soc.* **131**, 290 (1984).
